# Metastable Differentially Methylated Regions within Arabidopsis Inbred Populations Are Associated with Modified Expression of Non-Coding Transcripts

**DOI:** 10.1371/journal.pone.0045242

**Published:** 2012-09-20

**Authors:** Ericka R. Havecker, Laura M. Wallbridge, Paola Fedito, Thomas J. Hardcastle, David C. Baulcombe

**Affiliations:** 1 Department of Plant Sciences, University of Cambridge, Cambridge, United Kingdom; 2 BIOMAA, University Mediterranea of Reggio Calabria, Reggio Calabria, Italy; CNRS, France

## Abstract

Individual plants within a population may vary at both genetic and epigenetic levels. The rate of genetic divergence and its underlying mechanisms is well understood. Less is known about the factors contributing to epigenetic divergence among isogenic populations except that, despite the presence of mechanisms that faithfully maintain epigenetic marks, epigenetic differences are more frequent than genetic variation. Epigenetically divergent stretches of isogenic DNA sequence are called epialleles. Currently, it is not clear why certain regions exhibit variable epigenetic status. We identified and characterised two long RNA transcripts with altered expression and DNA methylation in an *ago5* mutant. However, further investigation revealed that these changes were not dependent upon AGO5. Rather, the variable transcription of these loci in *Arabidopsis* mutant and wild-type populations corresponds to spontaneous differential methylated regions (DMRs) or epialleles. These two DMRs are delineated by RNAs which are highly expressed when the DMR is hypomethylated. Furthermore, they control the expression of 5′ transcriptional start site mRNA variants of nearby protein coding genes. Our data support the recent observations that meiotically stable DMRs exist within inbred populations. We further demonstrate that DMR boundaries can be defined by putative non-coding promoter-associated transcripts.

## Introduction

Epigenetic information is stored through chemical modification of DNA and its associated histones [1], [2]. These modifications may be targeted to the appropriate genomic regions through an ancient mechanism in eukaryotes in which small RNA (sRNA) molecules are the specificity determinant. The sRNAs are associated with proteins that cause DNA or chromatin to be epigenetically modified and, in many instances, transcriptionally silent [3], [4]. Paradoxically, transcription of RNAs (likely through non-coding RNAs) is required for this transcriptional silencing process [5], [6], [7].

In *Arabidopsis thaliana*, the sRNAs associated with epigenetic modification and transcriptional gene silencing (TGS) are 24 nt long [8]. The 24 nt RNA silencing pathway involves a POL IV complex in which the largest subunit is NRPD1. POLIV transcribes a non-coding RNA that is made double-stranded (ds) by RNA-dependent RNA polymerase 2 (RDR2). This dsRNA is then cleaved into 23–24 nt sRNAs by Dicer-like 3 (DCL3). The 24 nt sRNA duplexes associate with an Argonaute (typically AGO4) and target it to non-coding RNA scaffold transcripts produced either by a POLV complex in which the largest subunit is NRPE1 [5], [6], [7], [9] or by POL II [10]. Targeting of the scaffold transcripts by AGO4 leads to recruitment of the DNA methyltransferase DRM2 and *de novo* cytosine methylation [9]. This process is referred to as RNA-directed DNA methylation (RdDM).

Once initiated, RdDM can be maintained through DNA replication independently of these RNA silencing components if the methylated cytosine is in a symmetrical context. The DNA methyltransferase MET1 maintains CpG methylation while CMT3 maintains CpHpG methylation [11]. However, for maintenance of methylated cytosine residues in an asymmetric context and, in some instances, at CpHpG motifs, the POLIV/POLV 24 nt sRNA silencing pathway is required [11].

The gain or loss of methylated cytosines [12] generates epialleles or differentially methylated regions (DMRs). Epialleles are phenotypically distinct if the change is in or close to a coding or regulatory locus [13], [14], [15], [16] and, together with changes in DNA sequence, they contribute to the heritable variation between members of populations. Stable epialleles can be generated by inbreeding of *met1* or *ddm1* mutants in which CpG methylation is defective [17], [18], [19]. Epialleles can also occur spontaneously [12]. Such epigenetic changes in *A. thaliana* are much more frequently than changes in DNA sequence [20], [21].

In this study, we identified two long RNAs that were mis-regulated in an *ago5* mutant. Mis-regulation of these loci in the *ago5* line was associated with differential DNA methylation. However, these RNAs were not mis-regulated because of a loss of AGO5. Instead they correspond to meiotically stable DMRs or epialleles that exist in random SALK populations and amongst natural *A. thaliana* ecotypes as well as in other RNA silencing mutants. Methylation maintenance at these loci is controlled by MET1 independently of RNA silencing. The epigenetic status of these DMRs affected their associated non-coding RNAs as well as specific mRNA variants of nearby protein coding genes.

Our results confirm two recent studies indicating that genetically identical individuals from a single population can be epigenetically divergent and that the epigenetic status of *A. thaliana* populations can change in relatively short time periods [20], [21]. Furthermore, our characterization of these two DMRs suggests that transcripts in promoter regions may promote epiallele formation. Our findings are relevant to understanding heritable variation in plant populations, and they also carry a cautionary message for those using mutants to investigate epigenetic effects: detailed controls should be used to differentiate spontaneous epigenetic changes from those due to any mutant in the machinery of epigenetics.

## Results

### Identification of Differentially Methylated Regions in the DNA of RNA Silencing Mutants

The initial aim of our study was to assign a biological role to AGO5 by analysis of long RNA transcripts that are mis-regulated in *ago5* mutants. However, there was no overlap between the top 200 mis-regulated transcripts (Figure S1A) and AGO5 associated sRNAs (data not shown). To investigate this mis-regulation we analyzed in detail the two loci with the largest RNA abundance changes in *ago5-2* compared with Col-0.

The RNA with the largest change was more abundant in *ago5-2* than in Col-0 and was derived from an un-annotated region near Chr1∶24080015 (this transcript was renamed *RITA* for RNA In The Antisense orientation) (Figure S1B). The tag with the second largest abundance change was, in contrast, reduced in *ago5-2*, and matched *Methionine Responding Down 1* (*MRD1*/*At1g53480*), a transcript described to be down-regulated in mutant *mto1* [22] that over-accumulates soluble methionine (Figure S1B).

To find out whether the change in methylation and transcription at *RITA* and *MRD1* is linked to RdDM we analyzed these loci in *ago4-3* as well as in Col-0, *ago5-2*. An McrBC assay indicated a loss of DNA methylation in *ago5-2* and *ago4-3* at *RITA* and an increase in methylation at *MRD1* relative to Col-0 (Figure S1C). In these assays, high levels of DNA methylation corresponded to low RNA abundance. We also assessed the transcriptional and methylation status of these loci in other RNA silencing mutants in: *AGO1*, *AGO2*, *AGO3*, *AGO4*, *AGO5*, *AGO6*, *AGO7*, *AGO8*, *AGO9*, *AGO10*, *RDR1*, *RDR2*, *RDR6*, *SGS3*, *DCL4*, *AtTEX1*, *SDE5*, *DCL1*, *JMJ14*, *NRPD1*, *NRPE1*, and *DCL3*.

**Figure 1:**
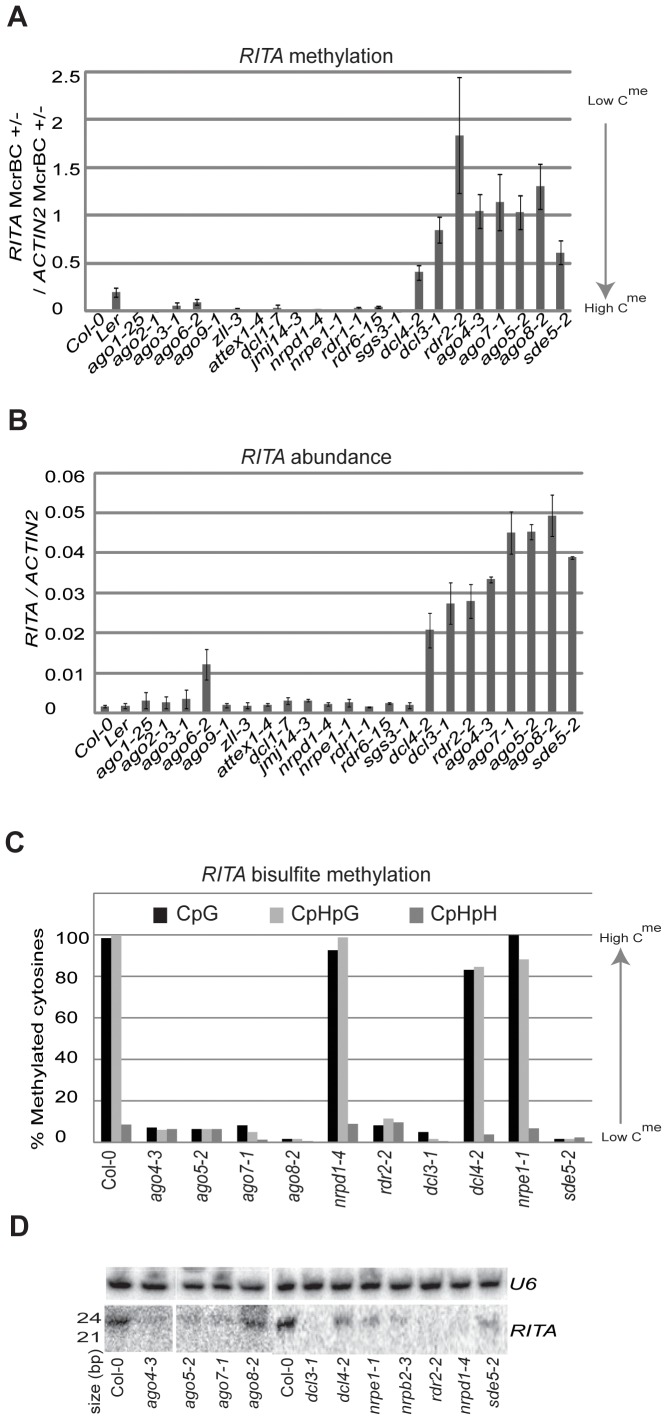
*RITA* characterization. (**a**) Methylation of *RITA* in seedlings as estimated by McrBC digestion in a panel of mutants. (**b**) *RITA* transcript abundance in seedling in a panel of mutants. (**c**) Bisulfite sequencing of *RITA*. Y-axis indicates percent of methylated cytosines. (**d**) sRNA northern blot using *RITA* probe. *U6* is a loading control.

At *RITA* there was loss of DNA methylation in plants lacking *DCL4*, *DCL3*, *RDR2*, *AGO4*, *AGO7*, *AGO5*, *AGO8* and *SDE5* (Figure 1A). As in *ago5-2* and *ago4-3*, there was a negative correlation of long RNA abundance and DNA methylation phenotypes in the different mutants: lower levels of *RITA* DNA methylation corresponded to increased long RNA (Figures 1B). *RITA* (referred to as BT025685) was also previously identified as a non-coding RNA increased in *hyl1*, but not in *dcl1* or *serrate* [23], suggesting other RNA silencing proteins might affect its epigenetic status.

However the DNA methylation changes did not have either of two hallmarks of RdDM. First the bisulfite DNA sequencing of *RITA* in Col-0, *ago5-2, ago4-3*, *nrpd1-4*, *nrpe1-1*, *rdr2-2*, *dcl3-1*, *dcl4-2*, *ago8-2*, *ago7-1* and *sde5-2* confirmed hypomethylation but only at cytosine residues in CpG and CpHpG contexts (Figure 1C). Second there was no correlation of DNA methylation with 24 nt sRNAs. These RNAs were absent in *nrpd1-4* in which there was no loss of DNA methylation and in *rdr2-2* and *dcl3-1* in which there was. These sRNAs were also reduced in other mutants but without correlation to the DNA methylation status at *RITA* (Figure 1D). Based on these analyses we conclude that the variable expression of *RITA* is due to spontaneous methylation changes at the DNA of these loci rather than the effects of the individual mutations.

**Figure 2:**
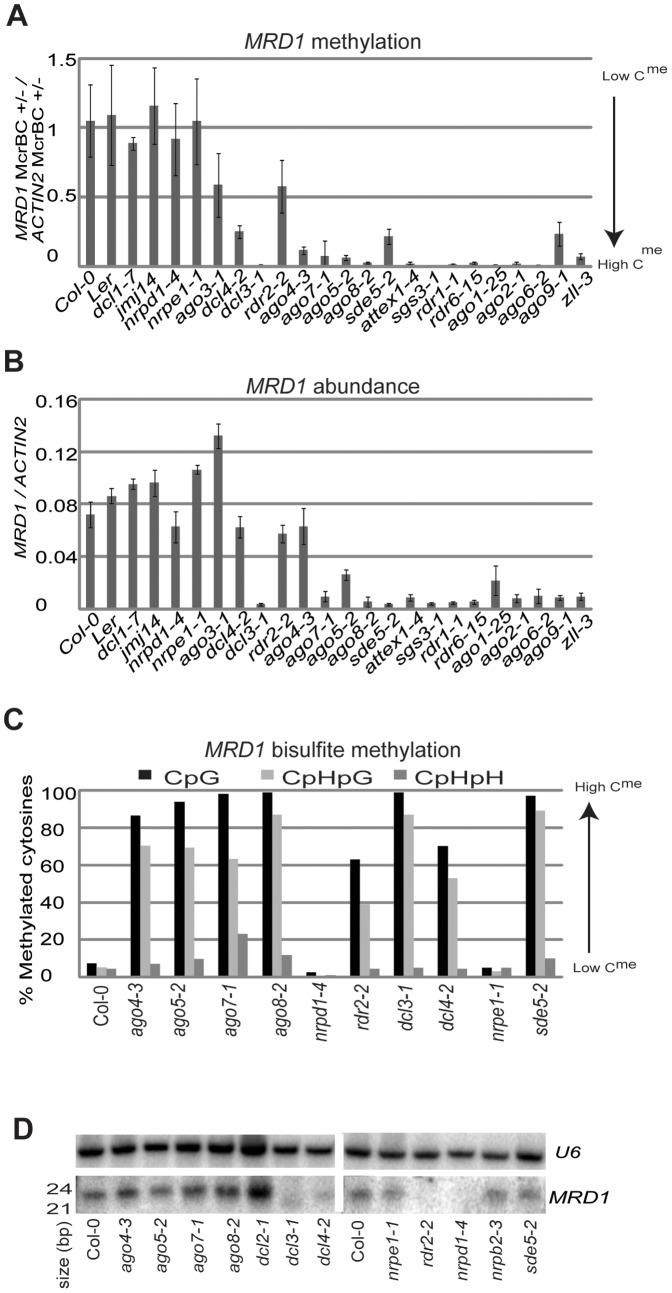
*MRD1* characterization. (**a**) Methylation of *MRD1* in seedlings as estimated by McrBC digestion in a panel of mutants. (**b**) *MRD1* transcript abundance in seedling in a panel of mutants. (**c**) Bisulfite sequencing of *MRD1*. Y-axis indicates percent of methylated cytosines. (**d**) sRNA northern blot using *MRD1* probe. *U6* is a loading control.

A similar spontaneous change in DNA methylation is also likely to explain the variation at *MRD1.* Two recent studies identified the *MRD1* locus as a variable DMR [20], [21] and in addition to the variation in expression and DNA methylation of this locus in Col-0 and *ago5-2* (Table S1, Figures S1B and S1C) there were inversely correlated changes in other mutants. Thus the *MRD1* DNA was hypomethylated and *MRD1* RNA was abundant in Col-0 and plants mutant for DCL1, JMJ14, NRPD1, NRPE1, RDR2 and AGO3 whereas the DNA was hypermethylated relative to Col-0 (Figure 2A) and RNA was present at a low level in other lines (Figure 2B). Bisulfite sequencing of lines with different *MRD1* states confirmed our McrBC assay and also that DNA methylation changes were at CpG and CpHpG contexts (Figure 2C). Thus, like *RITA*, the RNA abundance of *MRD1* correlated with its epigenetic state (Figure 2B). This correlation, as with *RITA*, was not correlated with sRNA: the *MRD1* 24 nt sRNAs were reduced in plants that were mutant for *NRPD1*, *RDR2*, *DCL3* and *DCL4* (Figure 2D) in which there were variable levels of *MRD1* DNA methylation and RNA.

### *RITA* and *MRD1* are DMRs Independent of RNA Silencing Proteins

**Figure 3:**
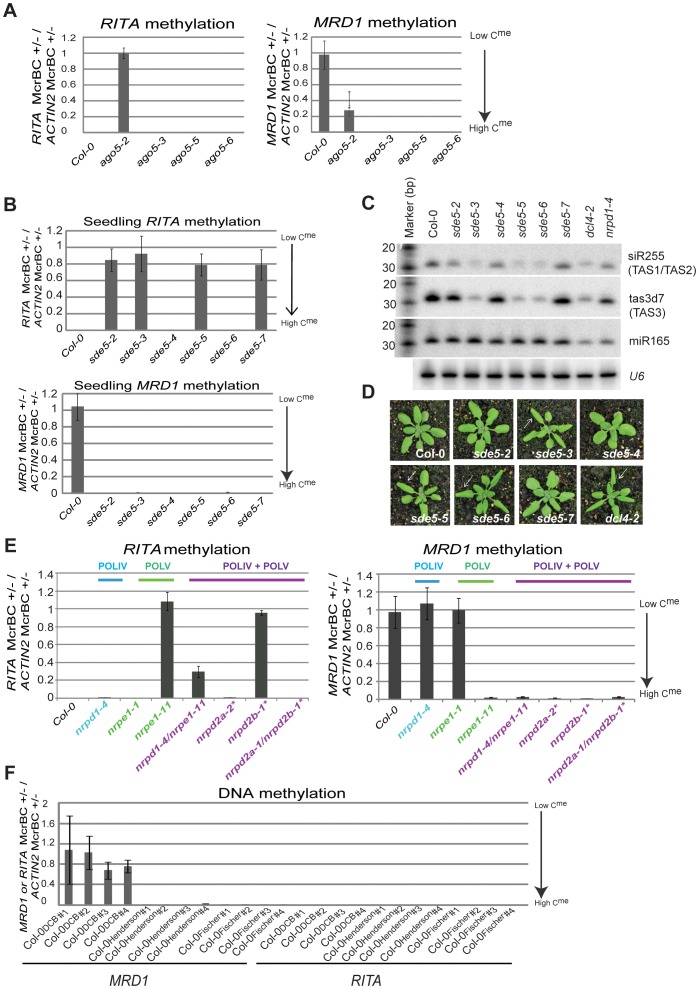
*RITA* and *MRD1* epigenetic statuses in various allelic series. (**a**) Methylation (estimated by McrBC digestion) of *RITA* and *MRD1* in the *AGO5* allelic series. (**b**) McrBC estimation of the methylation levels of *RITA* and *MRD1* in an *SDE5* allelic series. (**c**) sRNA northern blot for *SDE5* allelic series. miR165 and *U6* are loading controls. (**d**) *SDE5* allelic series mutant phenotypes. White arrows highlight accelerated juvenile-adult transition phenotype in leaves. (**e**) Methylation of *RITA* and *MRD1* in seedling RNA polymerase mutant alleles. The lines indicated with a * are also known to be mutants in the POLV complex (NRPE). (**f**) DNA methylation of *RITA* and *MRD1* in Col-0 lines obtained from different lab (denoted by the principal investigator’s surname). #’s refer to individual plants; 4 individuals were assayed per line.

The independence of DNA methylation and sRNA accumulation was further reinforced by the analysis of *RITA* in different alleles of *AGO5*, *SDE5*, *DCL2*, *AGO8* and *POLV*. In all members of an *ago5* allelic series (Figure S2A) there were reduced levels of AGO5 protein relative to Col-0 (Figure S2B) but *RITA* was hypomethylated only in *ago5-2* (Figure 3A). Similarly with alleles of *SDE5*, an RNA silencing protein implicated in the trans-acting sRNA pathway rather than the RdDM pathway [24], [25] there was hypomethylation of *RITA* DNA in *sde5-2, sde5-3*, *sde5-5* and *sde5-7* (Figure 3B) but not *sde5-4* and *sde5-6*. Of these mutants the strongest loss of function, manifested as accelerated juvenile-adult transition in leaves and loss of TAS1, TAS2 and TAS3 transacting sRNAs was in *sde5-3* and *sde5-5* in which there was hypomethylation of *RITA* DNA and in *sde5-6* in which *RITA* DNA was fully methylated (Figure 3C–3D).

Similarly, we assayed three independent T-DNA insertion mutants of AGO8 exons of which only *ago8-2* showed *RITA* hypomethylation (Figure S3A). All three mutants had inserts in exons and are likely to have lost AGO8 function. Additionally, in a *DCL2* allelic series, a *dcl2-1* line but not *dcl2-2* or *dcl2-3* had hypomethylated *RITA* (Figure S3B). Finally, we assayed two mutant alleles of the largest subunit of POLV (*nrpe1-1* and *nrpe1-11*), the *nrpd1-4 nrpe1-11* double mutant and multiple alleles of *nrpd2a,* the second largest subunit for POLIV and POLV (Figure 3E). As with the other mutants there was no correlation of strong loss of RNA silencing function and the hypomethylated *RITA* DNA.

In a similar analysis of DNA methylation at *MRD1* we tested alleles of *AGO5, SDE5, AGO8, DCL2* and *NRPE1*. Most of these mutants were hypermethylated at *MRD1* relative to Col-0 (Figure 3A–D, Figure S3A-B). However, *MRD1* DNA was hypomethylated in *nrpe1-1* and hypermethylated relative to Col-0 in *nrpe1-11* (Figure 3E). Thus the methylation status of *MRD1* DNA is independent of the RNA silencing machinery.

Combined, these results strongly suggest that *RITA* and *MRD1* are DMRs or epialleles and that the different epigenetic states in RNA silencing mutants are due to spontaneous variation. In support of this hypothesis, *MRD1* (but not *RITA*) was identified as being a variable DMR over 30 generations of of individual lineages [20], [21] and its epigenetic state varied in Col-0 stocks from different labs. This locus is hypomethylated in the Col-0_Baulcombe_ line but hypermethylated in Col-0_Henderson_ and Col-0_Fisher_ lines (Figure 3F). Similarly there was epigenetic variation at both *MRD1* and *RITA* in randomly selected SALK mutant populations and in *A. thaliana* ecotype populations. Fourteen randomly selected SALK lines along with Columbia based ecotypes (Col-0, Col-1, Col-2, Col-4, Col-6) demonstrated variability in the epigenetic status of both *RITA* and *MRD1* (Figure S4A-B).

We did not detect variable methylation of *RITA* DNA in Col-0_Baulcombe_ and in five natural ecotype populations (C24, Kas, Koud, Sha, Ws-0). We were unable to amplify *RITA* with our standard primers in Bay-0 and Na-1 ecotypes suggesting sequence divergence in these lines. By comparison, *MRD1* was hypomethylated in Col-0_Baulcombe_, C24, Koud and Sha ecotypes hypermethylated in Kas, Ws-0, Bay-0 and Na-1 (Figure S4C-D). Thus the DMR at *MRD1* is more variable in natural ecotypes than the DMR at *RITA*.

### DNA Methylation at *RITA* and *MRD1* is Controlled by MET1

**Figure 4:**
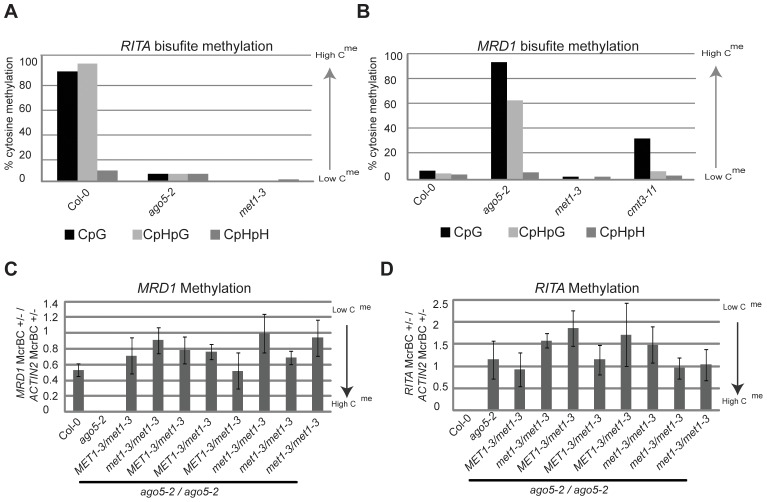
Role of MET1 on DMRs. (**a**) Bisulfite sequencing for *RITA* in *ago5-2* and *met1-3* mutants. (**b**) Bisulfite sequencing for *MRD1* in *ago5-2*, *met1-3* and *cmt3-11* mutants. (**c**) McrBC analysis for *RITA* methylation in *ago5-2*, *met1-3* heterozygotes and *met1-3* heterozygotes and these *met1* mutants combined with *ago5-2*. Each column represents a pool of individuals from separate populations. (**d**) McrBC analysis for *MRD1* methylation in *ago5-2*, *met1-3* heterozygotes and *met1-3* heterozygotes and mutants combined with *ago5-2*. Each column represents a pool of individuals from separate populations.

Bisulfite sequencing indicated that the levels of CpG and CpHpG DNA methylation can approach 100% at *RITA* and *MRD1* alleles but in *met1-3* methylation is almost completely lost (Figure 4A–B). This result confirms previous data and suggests that, at these loci, MET1 maintains both CpG and CpHpG methylation [26]. Alternatively it could be that direct maintenance of CpG methylation by MET1 is indirectly required for CpHpG methylation.

In order to confirm that MET1 controls the epigenetic status of *MRD1*, we crossed a *met1-3* line with hypomethylated *MRD1* to a line with hypermethylated *MRD1* (*ago5-2*). Three F4 populations were assayed for their MRD1 methylation status in various mutant genotypes. In all 3 populations, the *met1-3/met1-3* plants were hypomethylated at *MRD1* (Figure 4C). Thus, MET1 is likely to be required for maintaining high methylation at *MRD1* epialleles. However, in the *MET1*/*met1-3* genotypes, *MRD1* and *RITA* are similarly hypomethylated, suggesting that MET1 cannot re-establish methylation at these loci by itself. We cannot rule out that a hypermethylated *MRD1* epiallele segregated away in all 3 populations, but the most likely explanation is that MET1 is required for the maintenance of methylation at these loci and an additional factor is involved in remethylation (Figure 4C-D).

### Stable Inheritance of *RITA* and *MRD1*

We assessed the meiotic stability of DNA methylation at *RITA* and *MRD1* by using reciprocal backcrosses between Col-0 and *ago5-2*, and between Col-0 and *ago4-3* in which the parents differed in their epigenetic status of *RITA* and *MRD1*. Without regard to direction of the cross, the McrBC assay indicated the F1 progeny were 50% methylated at *RITA* and *MRD1* (Figure S5A-B). Thus, neither a functional copy of AGO5 nor AGO4 could remethylate *MRD1* and *RITA*. This was in contrast to the canonical POLIV locus *AtSN1,* which was remethylated in F1 progeny of *ago4-3* and Col-0 (Figure S5C).

We hypothesized that 50% methylation in the F1 progeny was an additive effect resulting from inheritance of one methylated and one unmethylated epi-allele from each parent. In order to differentiate the parental alleles for these DMRs, we crossed a line with hypomethylated *RITA* (Col-0-derived *ago5-2*) to a line with hypermethylated *RITA* (Ler with an insert at *RITA* relative to Col-0). The McrBC assay in the F1 progeny allowed us to visualize the methylation status of and differentiate between parental *RITA* alleles. In the F1 progeny of *ago5-2* and Ler, the Ler allele retained methylation whereas *RITA* inherited from *ago5-2* retained its hypomethylated state (Figure S5D). Thus, the epigenetic status of *RITA* is inherited through meiosis.

### DMRs and POL II Transcripts from *RITA* and *MRD1*

The methylation status of *RITA* and *MRD1* DNA may influence transcription of their nearby protein-coding genes as do the epialleles of *FWA* or *SUPERMAN* [14], [27] (Table S1, Figures S1B, 1, 2). To investigate this possibility we used 5′ and 3′ RACE to characterise *RITA* and *MRD1* transcripts in lines with the different epialleles at these loci.

**Figure 5:**
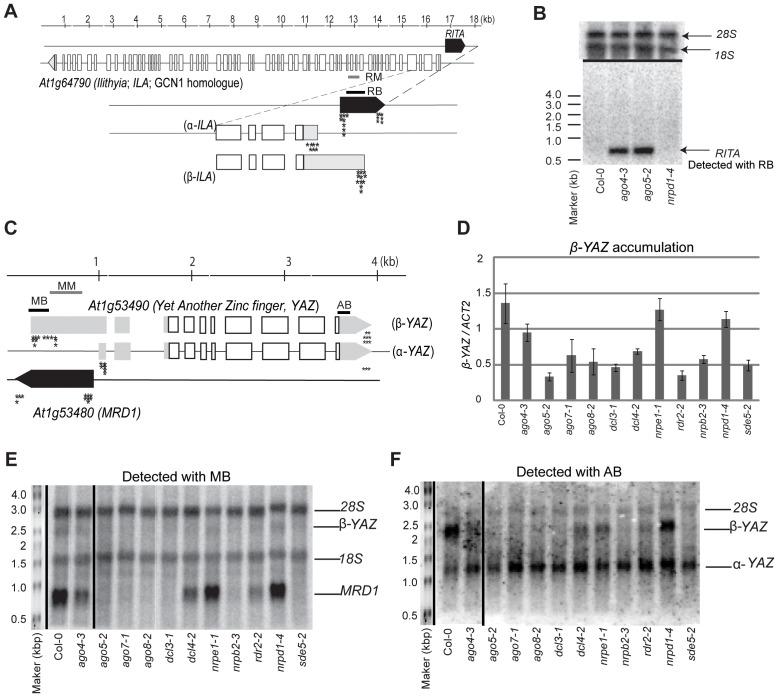
Analysis of *RITA* and *MRD1* locus transcripts. (**a**) Schematic of *RITA/ILA* locus. Stars indicate the location of individual 5′ and 3′ RACE clones. A ruler present is present above to estimate sizes. Open boxes indicate coding regions, gray shading indicated untranslated regions (UTRs) and black shading indicates *RITA* or *MRD1* RNAs. RM is the region assayed by bisulfite sequencing with oligos DBO489 and DBO490. RB is the region covered by McrBC assay using the oligos DBO447 and DBO448. MB was also used to probe the long RNA northern in (b). (**b**) Long RNA northern probed for *RITA*. *18S* and *28S* ribosomal RNAs indicate loading. (**c**) Schematic of *MRD1/YAZ* locus indicating amplicons (MB and AB) used for long RNA northern blot probes. MB was used for the McrBC assay with oligos DBO547 and DBO762. MM refers to the region assayed by bisulfite sequencing with the oligos DBO600 and DBO601. Stars indicate the location of individual 5′ and 3′ RACE clones. A ruler present is present above to estimate sizes. Open boxes indicate coding regions, gray shading indicated UTRs and black shading indicates non-coding RNAs. (**d**) RT-PCR for *β-YAZ* accumulation normalized to *ACT2*. (**e**) Long RNA northern blot with a probe (MB) that detects *β -YAZ* and *MRD1*, along with the non-specific hybridization of *18S* and *28S* ribosomal RNAs. (**f**) Long RNA northern blot with a probe (AB) that detects *β -YAZ* and *α-YAZ*. Loading is shown by *18S* and *28S* ribosomal RNAs in (e).

*RITA* generates a 687 base pair (bp) transcript that corresponds to a partial EST clone BT025685 and is divergently transcribed from *ILA* (Figure 5A). Northern analyses confirmed the size of the *RITA* RNA and its increased abundance in lines with hypomethylated *RITA* DNA (*ago4-3* and *ago5-2*) relative to a line in which *RITA* DNA is hypermethylated (*nrpd1-4*) (Figure 5B). Two *ILA* transcripts were identified that differed in their 5′ transcriptional start site (Figure 5A). The longer *ILA* transcript (designated as β-*ILA*) begins within and overlaps *RITA*, while the shorter *ILA* transcript (designated as α-ILA) is separated from *RITA* by 165 bp.

The *RITA* transcript has two open reading frames (ORFs) encoding short proteins of 12 and 78 amino acids that do not have homologues in the genomes of any sequenced organism. It is likely therefore that *RITA* is a non-coding RNA (Figure S6A). In contrast, *ILA* is a large protein-coding gene required for embryogenesis and plant innate immunity. The encoded protein is likely to be the *Arabidopsis* GCN1 homolog [28], [29].

To determine whether the status of *RITA* influenced the adjacent *ILA* locus, we used nested RT-PCR to assay the combined *α+ β-ILA* transcripts or the *β*-*ILA* transcript alone (Figure S7A). The abundance of the combined *α+ β-ILA RNA* did not differ whether *RITA* was hypo- or hyper-methylated (Figure S7B). However, the *β-ILA* RNA was more abundant than wild type in lines in which *RITA* DNA was hypomethylated (such as in *ago5*-2) (Figure S7B). It is likely therefore that the epigenetic state of *RITA* affects the expression of the overlapping *β-ILA* but not the non-overlapping *a-ILA* transcript.

Gene models for *MRD1* predict an overlap with *At1g53490* encoding an uncharacterized protein predicted to contain a DNA binding domain, which we renamed as *Yet Another Zinc finger* (*YAZ*). Previous reports identifying *MRD1* as a DMR also noted a transcriptional affect on the *At1g53490* gene [20], [21]. However, our RACE characterisation demonstrated that three transcripts are present at the *MRD1/YAZ* locus (Figure 5C). *MRD1* is an intron-less transcript of approximately 1,082 bp [22], whereas *YAZ* is divergently transcribed and has two mRNA variants that arise from different transcriptional start sites. The longer *YAZ* transcript (*β- YAZ*) coincides with the full-length EST clone BX814259 and it overlaps *MRD1* (Figure 5C). The transcriptional start site of the *α-YAZ* RNA is separated from that of *MRD1* by 43 bp.

Three predicted ORFs for the *MRD1* transcript encode peptides of 5 amino acids (ORF1), 32 amino acids (ORF2) and 120 amino acids (ORF3) (Figure S6B). BLAST searches using the protein sequence of ORF3 did not identify any conserved domains or similar proteins within plants and weak similarity with bacterial methylases (Figure S6B). Thus, the transcriptional configuration of the *MRD1* and *RITA* loci both have an RNA that is divergently transcribed from two adjacent mRNAs and that it overlaps the longer transcript.

To determine whether the epigenetic state of *MRD1* affected the adjacent *YAZ* locus we used northern analysis of lines with different *MRD1* epialleles (Figure 5D–F). Using a probe (MB) that detected both the *MRD1* transcript and *β-YAZ* it was clear that *β-YAZ* RNA accumulated at a lower level than *MRD1* RNA. Both species are less abundant in lines with methylated *MRD1* DNA (consisting of: *ago4-3, ago5-2, ago7-1, ago8-2, dcl3-1, dcl4-2, nrpb2-3, rdr2-2* and *sde5-2*) (Figure 5E). A second probe (AB) confirmed the inverse correlation of *β-YAZ* transcripts and DNA methylation but it indicated that *α-YAZ* is unaffected by the epigenetic status of *MRD1* (Figure 5F). The northern analysis of *β-YAZ* was confirmed by RT-PCR (Figure 5D).

Combined, these results indicate that *RITA* and *MRD1* are both DMRs in which DNA methylation has a highly localised silencing effect. The DMRs affect divergently transcribed RNAs but only if they overlap the methylated regions.

## Discussion

### DMR Variability within *Arabidopsis*

Two recent studies have shown that DMRs in *Arabidopsis* can vary over 30 generations of inbreeding [21], [20] independent of DNA sequence or known environmental stimuli. These studies identified the DMR status of *MRD1* but not of *RITA*. Other examples of DMRs or ‘pure’ epialleles in maize inbreds in the absence of associated genetic polymorphisms have also been recently documented [30]. Our findings suggest that the DMRs at *RITA* and *MRD1* are likely similarly due to such spontaneous loss or gain of DNA methylation amongst SALK T-DNA or other mutant lines and between Columbia-accessions (Figures 3, S3 and S4).

DNA methylation at these loci is tightly associated with transcriptional repression of adjacent and overlapping genes (Figures 2, 5 and S7) so that changes in the status of a DMR could influence the plant phenotype. Thus, DMRs could affect the fitness of plants in wild populations as well as influence crop breeding programmes. For example, plant phenotypes resulting from DMRs include the *peloric* epimutation affecting flower morphology in *Linaria* [13] and genetic incompatibility between Arabidopsis accessions due to the epigenetic state of the gene *AtFOLT* [31].

As demonstrated here, DMRs complicate analysis of plant genomes because epigenetic differences amongst inbred laboratory lines may be the consequence of spontaneous changes in DNA methylation rather than the effect of mutations (Figures 1–3, S3, and S4S4). In this light, it may be necessary to re-evaluate the role of RNA silencing at some DMRs. For example, the reported effects of *hyl1* on *RITA* [23] and of *nrpd1-3* on *MRD1* [32] could be the result of spontaneous epigenetic changes in those lines rather than mutant phenotypes. To differentiate whether epigenetic differences amongst lines are the result of spontaneous epialleles or a genetic mutation, the epigenetic lesion should be restored either a backcross or by complementation with a wild type gene.

As with other epialleles, the methylation status of *RITA* and *MRD1* correlates with control of their nearby protein coding genes. Interestingly, the influence of the methylated *MRD1* and *RITA* DMR had a strong effect on only one of the two detected nearby protein coding gene variants. Similarly at locus *At5g43500* (*ACT11*) an upstream DMR controlled only one mRNA isoform [20]. These observations suggest that the effect of a DMR on nearby protein coding regions is highly localised. Furthermore and similar to other epialleles, the methylated status of *MRD1* varied amongst natural populations of *Arabidopsis* [31], [33], [34].

### Control of Epigenetic Changes at DMRs

Why are some loci prone to differential methylation whereas others are not? It is unlikely that any single feature is responsible but it is striking that, at *RITA* and *MRD1*, the DMR boundaries correlate with long and potentially non-coding RNAs (Figure 5). The presence of POL II transcription within gene promoters is widespread in eukaryotes and it cannot explain the differential methylation by itself [35], [36], [37], [38], [39]. However it could be that the length of the non-coding transcript and/or its overlap with the transcribed region of the protein-coding gene contribute(s) to the metastable status of the DMRs. Alternatively, perhaps the transcription of the RNA can mediate the erasure of methylation marks in the promoter region and render the *RITA* and *MRD1* DMRs (more so than others) susceptible to hypo-methylation.

## Methods

### RNA Silencing Mutants & Growth Conditions

The following mutants are in the Col-0 genetic background. *ago1-25* [40], *ago2-1* (SALK_003380), *ago3-1* (SM_3_31520), *ago4-3* (WISC_338A06), *ago5-2* (SALK_118422), *ago5-1/5-3* (SALK_063806), *ago5-5* (SALK_123506), *ago5-6* (SALK_062912), *ago6-2* (SALK_031553), *ago7-1* (SALK_037458), *ago8-1* (SALK_139894), *ago8-2* (SALK_151983), *ago8-3* (SALK_060402), *ago9-1* (SALK_127358), *nrpd1-4* (SALK_083051), *nrpe1-1* [41], *nrpe1-11* (SALK_029919), *nrpd2a-2* (SALK_046208), *nrpd2b-1* (SALK_008535), *jmj14-3* [42], *rdr1-1* (SAIL_672F11), *rdr2-2* (SALK_059661), *rdr6-15* (SAIL_617), *attex1-4* (SALK_100012), *sgs3-1* [43], *dcl2-1* (SALK_064627), *dcl2-2* (SALK_123586), *dcl2-3* (SALK_095069), *dcl3-1* (SALK_005512), *dcl4-2* (GABI_160G05), *sde5-2* (SALK_020726), *sde5-3* (WISC_429G09), *sde5-4* [25], *sde5-5* (SALK_026595C), *sde5-6* (SALK_115496C), *sde5-7* (SALK_026511C), *met1-3* [44], *cmt3-11* (SALK_148381), and *nrpb2-3* [10]. *dcl1-7* [45] was introgressed into Col-0. ‘*dcl234*’ combines *dcl2-1*, *dcl3-1* and *dcl4-1*. The *nrpd2a-1/2b-1* mutant combines *nrpd2a-1* (SALK_095689) with *nrpd2b-1*. The *NRPD2a* and *NRPD2b* mutations affect both the POLIV and POLV complexes (and so are also considered *NRPE2a* and *NRPE2b*, respectively). *zll-3* [46] is in Ler.

The random collection of SALK lines and other Columbia based ecotype lines were obtained from the European Arabidopsis Stock Centre (NASC) [47]. Table S2 details where the insertion is predicted to be located. Zygosity of the T-DNA inserts was not confirmed. Some Col-0 lines were obtained from other laboratories and are abbreviated as Col-_0Henderson_ (Ian Henderson lab.) and Col-0_Fischer_ (Robert Fischer lab.). Col-0 from the David Baulcombe lab designated as Col-0_DCB_ or simply Col-0.

Genotyping primers for alleles characterised in this study are in Table S3. *ago5-2* wild-type (DBO368/DBO369), *ago5-2* mutant (DBO369/Lba1), *ago5-1*/*ago5-3* wild-type (DBO372/DBO373), *ago5-1/ago5-3* mutant (DBO373/Lba1), *ago5-5* wild-type (DBO368/DBO371), *ago5-5* mutant (DBO371/Lba1), *ago5-6* wild-type (SALK_062912LP/SALK_062912RP), *ago5-6* mutant (SALK_062912LP/Lbb1), *ago8-1* wild-type (DBO80/DBO136), *ago8-1* mutant (DBO80/LBa1), *ago8-2* wild-type (DBO137/DBO119), *ago8-2* mutant (DBO119/Lba1), *ago8-3* wild-type (DBO132/DBO137), *ago8-3* mutant (DBO137/LBa1), *sde5-2* wild-type (DBO827/DBO828), *sde5-2* mutant (DBO828/Lba1), *sde5-5* wild-type (DBO829/DBO830), *sde5-5* mutant (DBO830/Lba1), *sde5-6* wild-type (DBO827/DBO828), *sde5-6* mutant (DBO827/Lba1), *sde5-7* wild-type (DBO831/DBO832), and *sde5-7* mutant (DBO832/Lba1).

Mixed stage inflorescence tissue was collected from plants 6-7 weeks post germination grown at 20°C, 16 hr day lengths. Seedlings (collected 14 days post germination) were grown on ½MS, 21°C (light), 20°C (dark) in 16 hr days.

### DNA Analyses

DNA samples were prepared using Qiagen Puregene reagents. 30 mg tissue was incubated in 300 µl Cell Lysis Solution (No. 158906) at 65°C (60 min), followed by incubation at 37°C (15 min) with 6 µg RNaseA. 100 µl Protein Precipitation Solution (No. 158910) was added followed by centrifugation at 13,000×g (3 min). DNA was precipitated with 100% isopropanol, followed by centrifugation at 13,000×g (1 min). DNA was washed and resuspended in DNA Hydration Solution (No. 158914).

For bisulfite analyses, 500 ng DNA was converted using EZ DNA Methylation-Gold Kit (Zymo Research), using the ‘Alternative 1’ conversion methods. PCR conditions (100 ng converted DNA) were: GoTaq PCR buffer (Promega), 2.5 mM MgCl_2_, 0.25 mM dNTPs, 10% DMSO, 1 mM primers and 1.5 units Taq. Bisulfite primers were: *RITA* (DBO489/DBO490), *MRD1* (DBO600/DBO601), *AtSN1* (JP1821/JP1822), *AtREP2* (DBO411/DBO412), *Simplehat2* (DBO413/DBO414). Amplification conditions were 94°C, 3′, 40 cycles of 94°C, 30″, 55°C, 30″, 50°C, 30″ and 62°C, 1.5′. At least 12 colonies were sequenced per locus, except at *MRD1* where at least 24 colonies were sequenced because approximately one-half correspond to *At5g03090*. *At5g03090* has approximately 90% sequence homology to *MRD1* and was methylated in all samples analyzed.

Bisulfite converted sequences were aligned to an unconverted parent using ClustalX. Non-cytosine SNPs were used to differentiate *MRD1* from *At5g03090*. Evaluation of methylation was with Kismeth [48].

For the McrBC assay, 1000 ng of DNA were diluted in 12.5 µl of DNA Hydration Solution, 1X NEB Buffer 2, 10 µg BSA, 1 mM GTP, and 75.5 µl water. 500 ng was then digested at 37°C for 12 hrs with 20 units McrBC (New England Biolabs) or mock digested using 2 µl 50% glycerol. The reactions were inactivated at 65°C, 20 min. 20 ng digested DNA was used per quantitative PCR reaction. McrBC assay primers (Table S3) are: *RITA* (DBO447/DBO448), except for the random SALK assay in which the more robust pair of (DBO709/DBO448) was used for *RITA*, *MRD1* (DBO547/DBO762), *ACTIN2* (ACT2 FW/ACT2 REV), *AtSN1* (AtS15, AtSN1-F4), *At3g01345* (DBO986/DBO987), and *At5g24250* (DBO1028/DBO978).

All quantitative PCR reactions were performed using 1X SYBR Green (Sigma Aldrich) and 0.15 µM primers (annealing temperature 56°C). Three technical replicates were done for all samples and the quantity of PCR product estimated as 1/1.8?C(t).

For the McrBC assay, the gene-specific quantity of McrBC digested DNA was normalized to the quantity of mock digested DNA. This value was then normalized to the normalized value (digest/mock) of the unmethylated gene *ACTIN2*. Error was calculated by standard propagation of error. Biological replicates were done at least twice for all experiments (with the exception of the random SALK lines which were only assayed once) but only 1 experiment is represented in the figures. There were no discrepancies observed amongst biological replicates.

### RNA Analyses

Total RNA was isolated using Trizol (Invitrogen). cDNA synthesis was done using a polyT primer on 500 ng DNAse (Turbo DNAse system, Ambion) treated RNA using SuperScriptIII (Invitrogen). Primers (Table S3) for RT-PCR reactions are: *RITA* (DBO447/DBO448), *MRD1* (DBO762/DBO547), *ACTIN2* (ACT2FW/ACT2RV), *AGO5* (DBO372/DBO373), *β-ABE* (DBO637/DBO758), *α*+*β-ILA* (DBO487/DBO488), *β-ILA* was detected with a primary PCR amplification (27 cycles) using (DBO920/DBO882) followed by 50X dilution and a nested PCR reaction (1 µl of the dilution, 28 cycles, DBO735/DBO834). A negative control was used for the nested *β-ILA* assay with DBO920/DBO708, where DBO708 lies outside the 5′ end of *β-ILA*. For the additionally characterised DMRs, transcripts were detected with these primers: *α-At5g24240* (DBO1067/DBO1068), *β-At5g24240* (DBO976/DBO1068), *UBC9* (DMB175/DMB176), *At3g01345* (DBO1069/DBO1070), *At3g01340* (DBO1008/DBO1010).

5′ and 3′ RACE reactions were carried using Clontech SMART RACE system following manufacturer’s instruction with the exception of NEB Phusion used for PCR amplification. Transcript ends were mapped from Col-0 transcripts with these primers (Table S3): *α-ILA* (DBO712, 5′ RACE; DBO710, 3′ RACE), *MRD1* (DBO714, 5′ RACE; DBO713, 3′ RACE), *β-YAZ* (DBO728, 5′ RACE), (DBO714 primary PCR followed by DBO715, 3′ RACE), *α-YAZ* (DBO757, 5′ RACE; DBO730, 3′ RACE). *RITA* and β*-ILA* were mapped in *ago5-2* because they are expressed there; *RITA* (DBO708, 5′ RACE; DBO709, 3′ RACE) and β*-ILA* (DBO877 primary PCR followed by β*-ILA* nested, 5′ RACE). All PCR products were gel purified and cloned (pGEM Teasy) and at least 8 colonies were sequenced.

Long RNA northern blots were performed with polyA RNA (Micro PolyA Purist, Ambion). Long RNAs were separated in 1X MOPS as described (Northern Max Protocol, Ambion), followed by capillary transfer (Zeta-Probe Membrane, BIO-RAD). Probe MB was amplified using DBO762/DBO547, Probe AB with DBO765/DBO766 and *RITA* probe with DBO447/DBO448 from cDNA. PCR products were radioactively labelled (Rediprime II, Amersham). Hybridizations were at 68°C (PerfectHyb Plus, Sigma).

sRNA northerns were carried out as previously described [51]. Oligos (Table S3) were radioactively labelled and hybridized at 35°C (ULTRAhyb-Oligo, Ambion). *MRD1* was detected with the oligo mix DBO810, DBO901, DBO843 and DBO900. *RITA* was detected with a DNA probe amplified with DBO447/448 (Rediprime II, Amersham), and hybridized at 45°C (ULTRAhyb, Ambion). All northern blots were washed in 2X SSC 0.1% SDS.

### Antibody Generation, Immunoblotting and Immunoprecipitation

The AGO5 peptide H_2_N-SKEESKNTEVSETMSC-CONH_2_ was KLH conjugated and used to raise rabbit polyclonal antibodies and affinity purified (Eurogentec). AGO5 immunoblotting was done at 1∶1500 (α-AGO5) in 5% milk, followed by a 1∶10000 titer of HRP-conjugated goat-anti-rabbit secondary antibody (Santa Cruz) in 5% milk and detected using ECL PLUS (Amersham). AGO1 (VRKRRTDAPSEGGEGC) anti-peptide antibody and immunoblotting conditions were described previously [49], [50]. αACTIN (1∶1000, Affinity Bioreagents) and αGAPDH (1∶5000, Abcam) were used following manufacturer’s recommendations and appropriate HRP-conjugated secondary antibodies.

Proteins were extracted from mixed staged floral tissue by grinding in liquid nitrogen and homogenization in 3X (w:v) extraction buffer (20 mM Tris HCL pH 7.5, 5 mM MgCl_2_, 5mM DTT, 300mM NaCl, 0.1% NP-40, 1% plant protease inhibitor (Sigma)). Extract was centrifuged and visualized by immunoblotting using 6% acrylamide SDS PAGE gels.

The Col-0 and *ago5-2* EST Tag sequences were prepared using the Illumina Digital Gene Expression – Taq Profiling for Nla III following manufacturer’s instructions for polyA RNA. All libraries were sequenced on a Genome Analyzer I or II.

The sequence data discussed in this publication have been deposited in NCBI’s Gene Expression Omnibus and are accessible through the GEO Series accession number GSE30970 (http://www.ncbi.nlm.nih.gov/geo/query/acc.cgi?acc=GSE30970).

The accession numbers are: Col-0 floral sRNAs (GSM767710), *ago5-2* floral sRNAs (GSM767711), AGO5 IP (GSM767709), Col-0 EST tag replicate 1 (GSM767714), Col-0 EST tag replicate 2 (GSM767712), *ago5-2* EST tag replicate 1(GSM767715), *ago5-2* EST tag replicate 2 (GSM767713).

### Bioinformatic & Statistical Analyses

The Illumina sequence data were processed as described previously [51]. EST tags were ranked according to their likelihood of differential expression as evaluated by ‘baySeq’ Bioconductor package [51] between Col-0 and *ago5-2*. The top 200 differentially expressed tags were aligned to the *Arabidopsis* genome (TAIR 10) and annotated as overlapping protein-coding genes, non-coding RNAs, or transposons.

## Supporting Information

Figure S1 ***ago5*** **-2 EST tag sequencing identifies mis-regulated transcripts.** **(a)** Annotations for genome regions corresponding to the 200 EST tags most likely to be differentially represented between Col-0 and *ago5-2*. Combined percentages are greater than 100 because some tags correspond to more than 1 annotation. **(b)** Quantitative RT-PCR confirms EST tag data for some *ago5-2* mis-regulated RNAs. Sample names appear below the bottom panel. Genes or loci tested appear above each individual panel. Chromosome coordinates are given for the locus named *RITA*, which did not correspond to a known gene. **(c)** McrBC digest of genomic DNA followed by PCR amplification of affected loci confirmed to be differentially expressed in *ago5-2*. Tall bars indicate low levels of methylation and low bars indicate high levels of methylation. Sample names appear below the bottom panel and individual loci are given above each panel. Error bars represent a propagation of errors.
(PDF)

Figure S2 **AGO5 allelic series analysis.** **(a)** Schematic diagram of the *AGO5* gene (*At2g27880*). Boxes represent that *AGO5* transcript; exons (white), UTRs (shaded). Vertical lines indicate the location of transfer-DNA insertions. Each allele is marked above its representative triangle, and the association of the SALK line and description with the mutant is under the schematic. No phenotypic changes were observed in any of the lines. Images were taken at the same magnification approximately two weeks after germination. **(b)** Western blot using αAGO5. αAGO1 was used as a loading control.
(PDF)

Figure S3 ***RITA*** **and** ***MRD1*** **methylation in the** ***AGO8*** **and** ***DCL2*** **allelic series** **(a)** Three T-DNA insertions within the predicted *AGO8* (*At5g21030)* gene were identified. A schematic shows the location of each of the insertions (open boxes represent putative exons). McrBC digestion followed by PCR estimates the methylated status for *RITA* and *MRD1* in each of these lines. **(b)** McrBC digestion followed by PCR estimates the methylated status for *RITA* and *MRD1* in 3 different *DCL2* alleles along with the *dcl234* triple mutant, which harbours the *dcl2-1* mutation.
(PDF)

Figure S4 **Naturally occurring** ***RITA*** **and** ***MRD1*** **epialleles in** ***A. thaliana*** **mutant lines and ecotypes. (a)** McrBC digestion estimating *RITA* methylation in various Columbia based ecotypes and randomly chosen SALK T-DNA insertion lines. N##### refers to the NASC identifier for each line. Each column represents a pool of approximately 6 individual seedlings. **(b)** McrBC digestion estimating *MRD1* methylation in various Columbia based ecotypes and randomly chosen SALK T-DNA insertion lines. N##### refers to the NASC identifier for each line. Each column represents a pool of approximately 6 individual seedlings. **(c)** DNA methylation analysis of *RITA* epialleles in Col-0, C24, Kas, Koud, Sha and Ws-0 seedlings by McrBC digest. DNA methylation at *MRD1* for Col-0_DCB_ is also shown. Four individuals were analysed for each ecotype. **(e)** DNA methylation analysis of *MRD1* epialleles in Col-0, C24, Kas, Koud, Sha, Ws-0 Bay-0 and Na-1seedlings by McrBC digest. Four individuals were analysed for each ecotype.
(PDF)

Figure S5 **Inheritance of** ***RITA*** **and** ***MRD1*** **. (a)** McrBC digestion followed by PCR estimating the DNA methylation at *RITA* of in F1 progeny of crosses between Col-0 and *ago5-2* and Col-0 and *ago4-3*. **(b)** McrBC digestion followed by PCR estimating the DNA methylation at *MRD1* of in F1 progeny of crosses between Col-0 and *ago5-2* and Col-0 and *ago4-3*. **(c)** McrBC digestion followed by PCR estimating the DNA methylation at *AtSN1* of in F1 progeny of crosses between Col-0 and *ago5-2* and Col-0 and *ago4-3*. **(d)** McrBC digestion followed by PCR shows allele specific methylation in crosses between Ler and *ago5-2* and Col-0 and *ago5-2*.
(PDF)

Figure S6 **Coding predictions for** ***RITA*** **and** ***MRD1*** **. (a)** The *RITA* RNA transcript has two Open Reading Frames (ORFs). The predicted number of amino acids appears below the ORF. Predicted homologous proteins are listed for ORF2 with accession, description and E value. **(b)** The *MRD1* transcript contains 3 ORFs. The number of amino acids predicted for each ORF is listed below the schematic. Blast P results including accession, description, and E value are listed for each of the hits.
(PDF)

Figure S7 **Transcript abundance changes of** ***ILA*** **in mutants with various** ***RITA*** **epigenetic states**. **(a)** Locus structure of *ILA* and *RITA*. Upper panel depicts the orientation of *α* - and *β-ILA* relative to RITA. Lower panel is a schematic of the RNA transcripts present at the locus. Open boxes represent coding regions; gray boxes represent UTRs; *RITA* is depicted in black. Labelled arrows correspond to the oligos used in RT-PCR reaction shown in (b). **(b)** RT-PCR of *ILA* transcripts. *ACT2* and *α*+*β-ILA* (most likely to be composed of mainly *α -ILA*) as measured by DBO487/DBO488do not significantly differ amongst the RNA silencing mutants tested. A nested PCR reaction using DBO735/DBO844on a primary PCR reaction designed only to amplify *β-ILA* transcripts demonstrated an increase in *β-ILA* in the mutants where methylation is lost at *RITA*. No amplification was observed in a primary reaction (DBO920/DBO882) or when the oligos were present outside of the mapped *β-ILA* transcript region (DBO920/DBO708).
(PDF)

Table S1 (XLSX)

Table S2 (XLSX)

Table S3 (XLSX)
