# Supplementary figures and images for: Metastable Differentially Methylated Regions within Arabidopsis Inbred Populations Are Associated with Modified Expression of Non-Coding Transcripts
Source: PLoS One. 2012 Sep 20;7(9):e45242. doi: 10.1371/journal.pone.0045242 (PMC3447930; doi:10.1371/journal.pone.0045242)

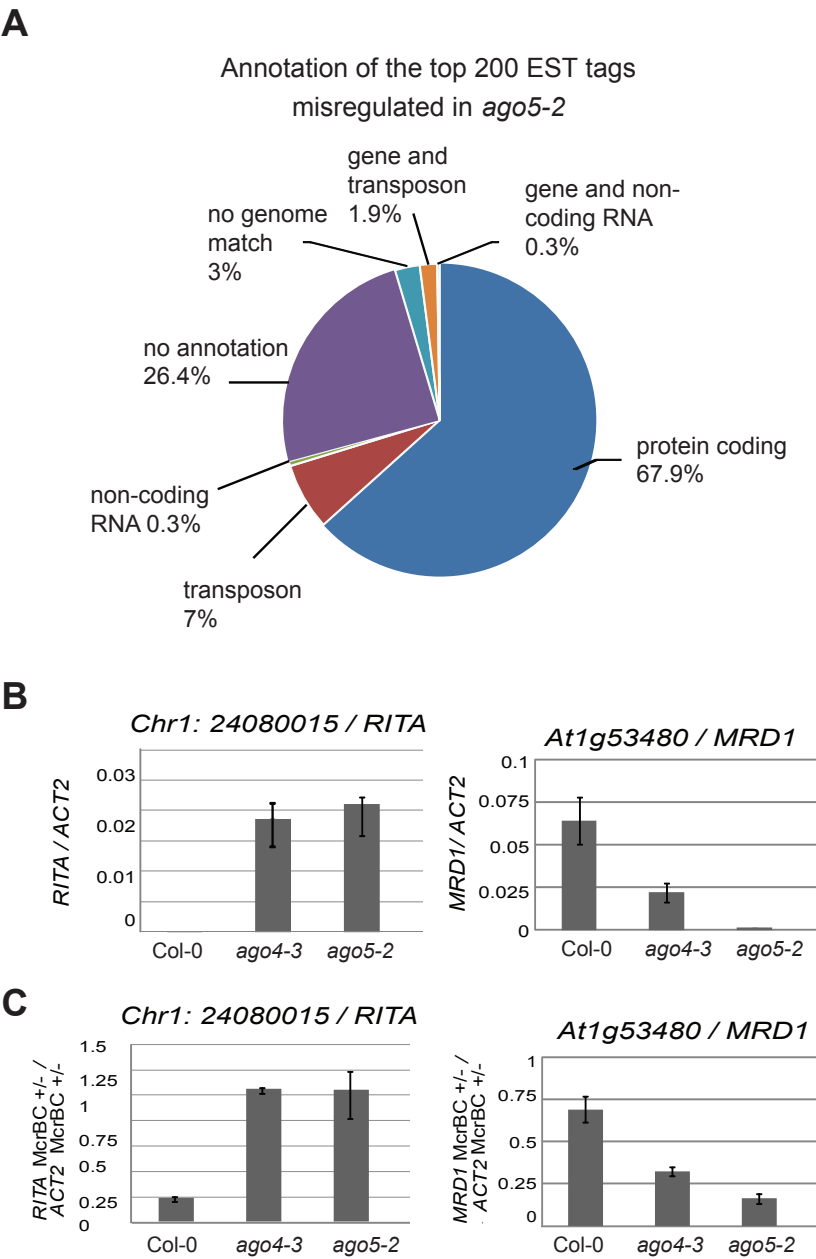

Supplement: Figure S1 — ago5 -2 EST tag sequencing identifies mis-regulated transcripts. (a) Annotations for genome regions corresponding to the 200 EST tags most likely to be differentially represented between Col-0 and ago5-2. Combined percentages are greater than 100 because some tags correspond to more than 1 annotation. (b) Quantitative RT-PCR confirms EST tag data for some ago5-2 mis-regulated RNAs. Sample names appear below the bottom panel. Genes or loci tested appear above each individual panel. Chromosome coordinates are given for the locus named RITA, which did not correspond to a known gene. (c) McrBC digest of genomic DNA followed by PCR amplification of affected loci confirmed to be differentially expressed in ago5-2. Tall bars indicate low levels of methylation and low bars indicate high levels of methylation. Sample names appear below the bottom panel and individual loci are given above each panel. Error bars represent a propagation of errors. (PDF) [file pone.0045242.s001.pdf]

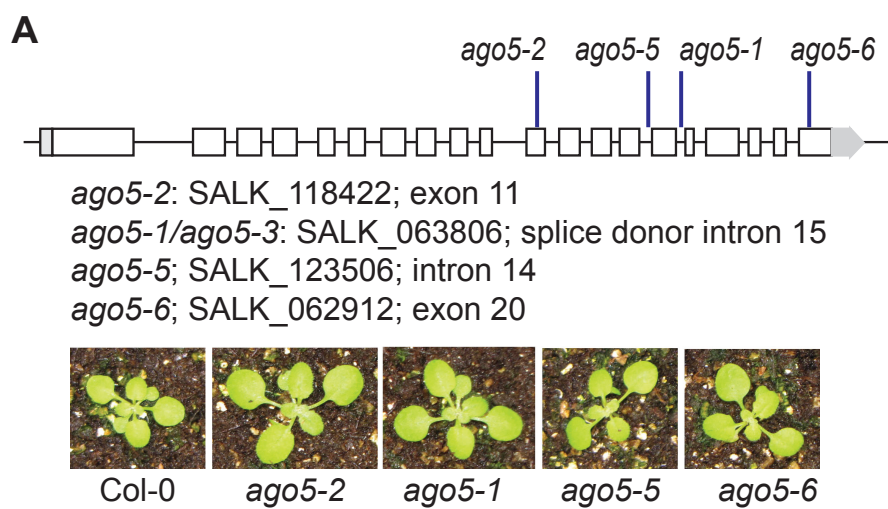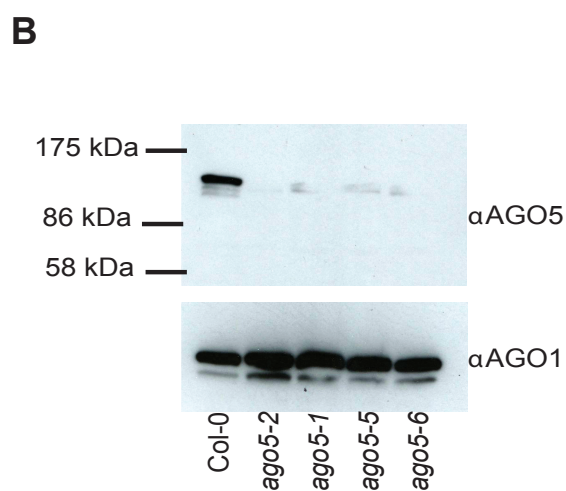

Supplement: Figure S2 — AGO5 allelic series analysis. (a) Schematic diagram of the AGO5 gene (At2g27880). Boxes represent that AGO5 transcript; exons (white), UTRs (shaded). Vertical lines indicate the location of transfer-DNA insertions. Each allele is marked above its representative triangle, and the association of the SALK line and description with the mutant is under the schematic. No phenotypic changes were observed in any of the lines. Images were taken at the same magnification approximately two weeks after germination. (b) Western blot using αAGO5. αAGO1 was used as a loading control. (PDF) [file pone.0045242.s002.pdf]

**A**

*AGO8 At5g21030*

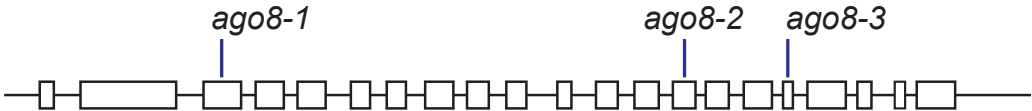

*ago8-1* SALK\_139894; predicted exon 3  
*ago8-2* SALK\_151983; predicted exon 14  
*ago8-3* SALK\_060402; predicted exon 17

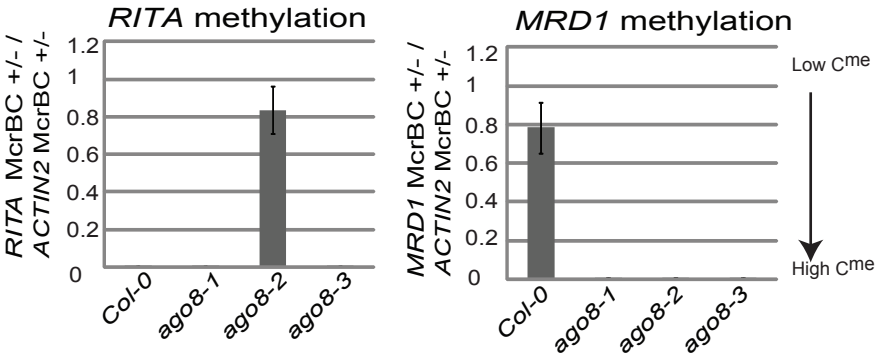

**B**

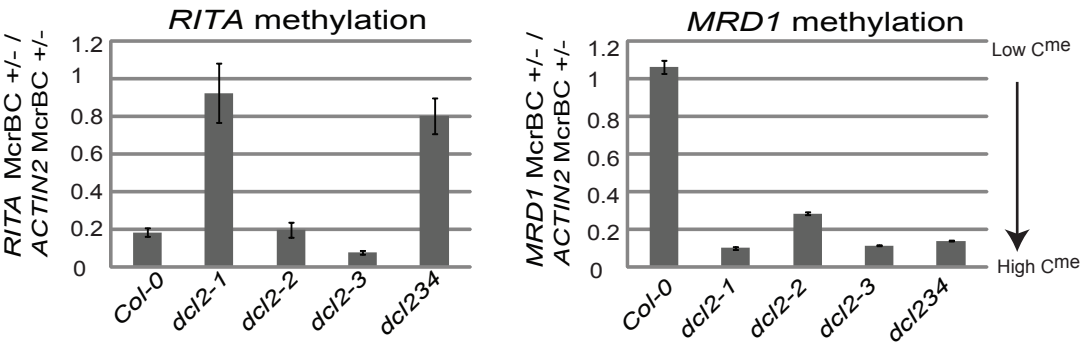

Supplement: Figure S3 — RITA and MRD1 methylation in the AGO8 and DCL2 allelic series (a) Three T-DNA insertions within the predicted AGO8 (At5g21030) gene were identified. A schematic shows the location of each of the insertions (open boxes represent putative exons). McrBC digestion followed by PCR estimates the methylated status for RITA and MRD1 in each of these lines. (b) McrBC digestion followed by PCR estimates the methylated status for RITA and MRD1 in 3 different DCL2 alleles along with the dcl234 triple mutant, which harbours the dcl2-1 mutation. (PDF) [file pone.0045242.s003.pdf]

Figure S4

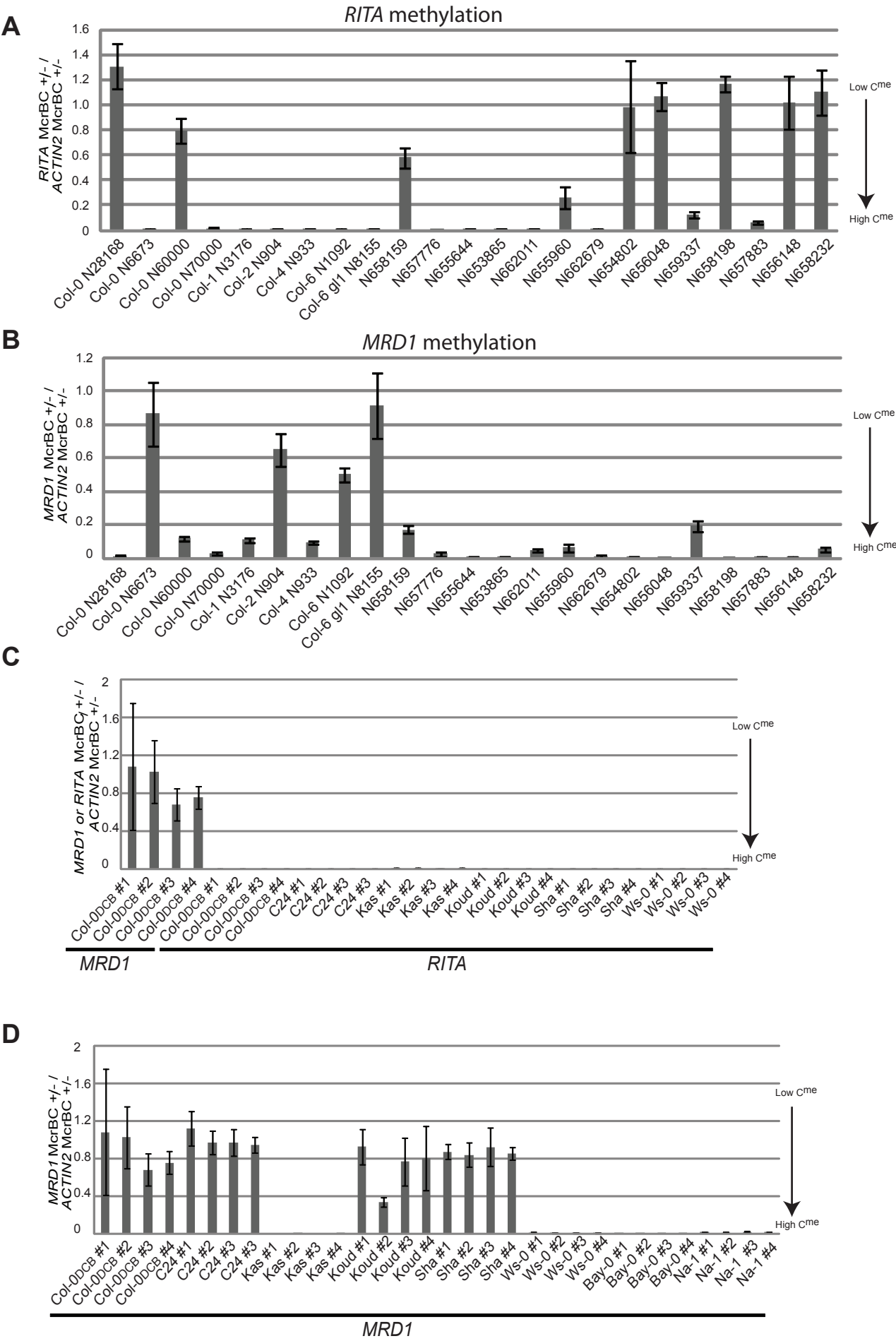

Supplement: Figure S4 — Naturally occurring RITA and MRD1 epialleles in A. thaliana mutant lines and ecotypes. (a) McrBC digestion estimating RITA methylation in various Columbia based ecotypes and randomly chosen SALK T-DNA insertion lines. N##### refers to the NASC identifier for each line. Each column represents a pool of approximately 6 individual seedlings. (b) McrBC digestion estimating MRD1 methylation in various Columbia based ecotypes and randomly chosen SALK T-DNA insertion lines. N##### refers to the NASC identifier for each line. Each column represents a pool of approximately 6 individual seedlings. (c) DNA methylation analysis of RITA epialleles in Col-0, C24, Kas, Koud, Sha and Ws-0 seedlings by McrBC digest. DNA methylation at MRD1 for Col-0DCB is also shown. Four individuals were analysed for each ecotype. (e) DNA methylation analysis of MRD1 epialleles in Col-0, C24, Kas, Koud, Sha, Ws-0 Bay-0 and Na-1seedlings by McrBC digest. Four individuals were analysed for each ecotype. (PDF) [file pone.0045242.s004.pdf]

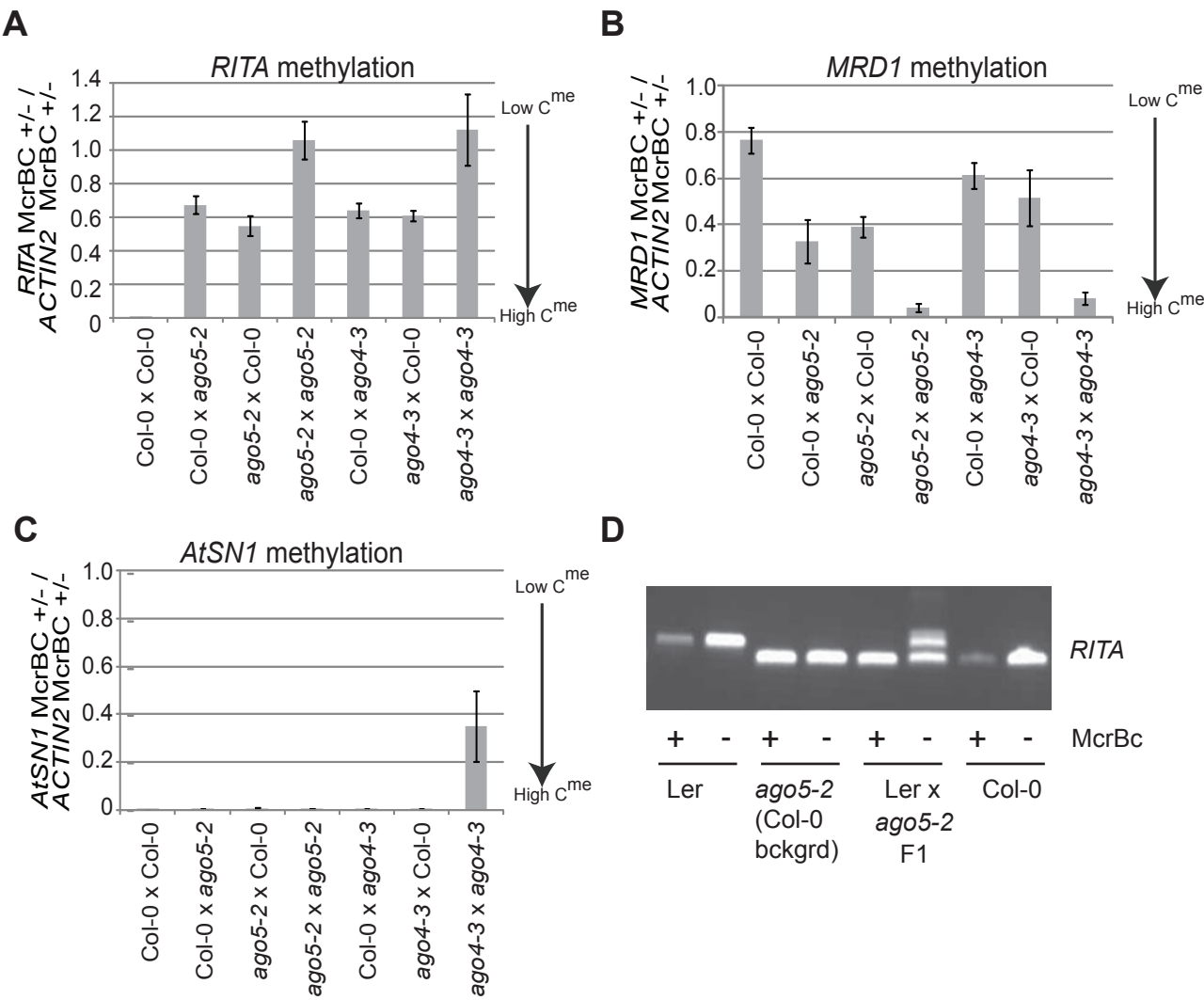

Supplement: Figure S5 — Inheritance of RITA and MRD1 . (a) McrBC digestion followed by PCR estimating the DNA methylation at RITA of in F1 progeny of crosses between Col-0 and ago5-2 and Col-0 and ago4-3. (b) McrBC digestion followed by PCR estimating the DNA methylation at MRD1 of in F1 progeny of crosses between Col-0 and ago5-2 and Col-0 and ago4-3. (c) McrBC digestion followed by PCR estimating the DNA methylation at AtSN1 of in F1 progeny of crosses between Col-0 and ago5-2 and Col-0 and ago4-3. (d) McrBC digestion followed by PCR shows allele specific methylation in crosses between Ler and ago5-2 and Col-0 and ago5-2. (PDF) [file pone.0045242.s005.pdf]

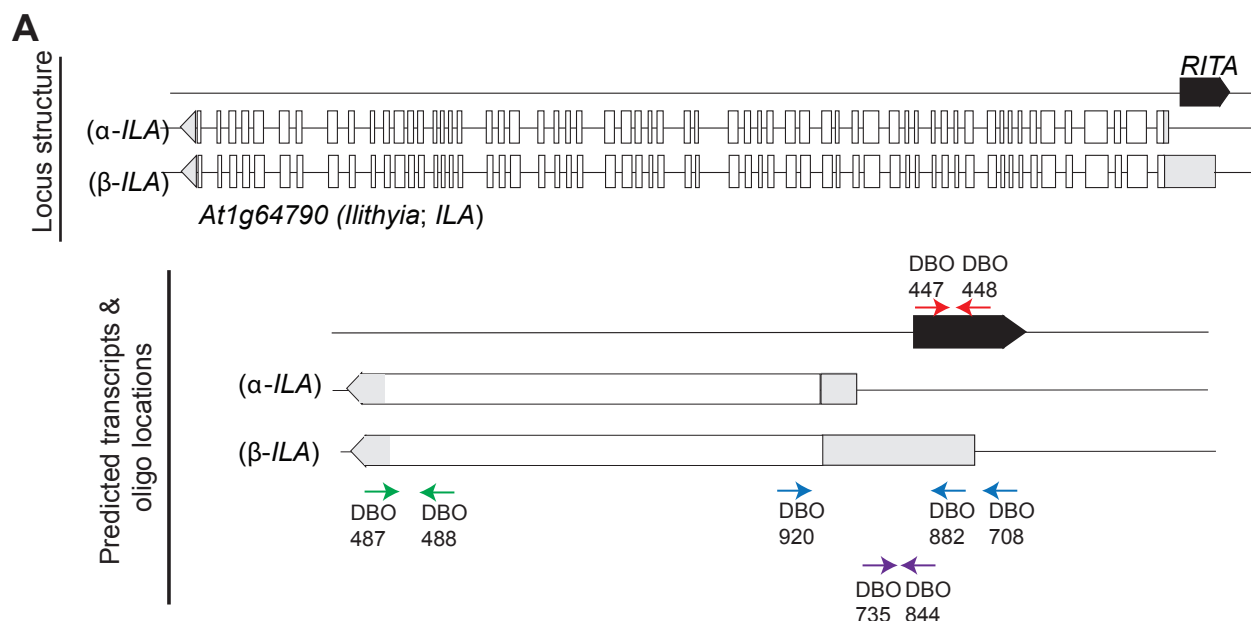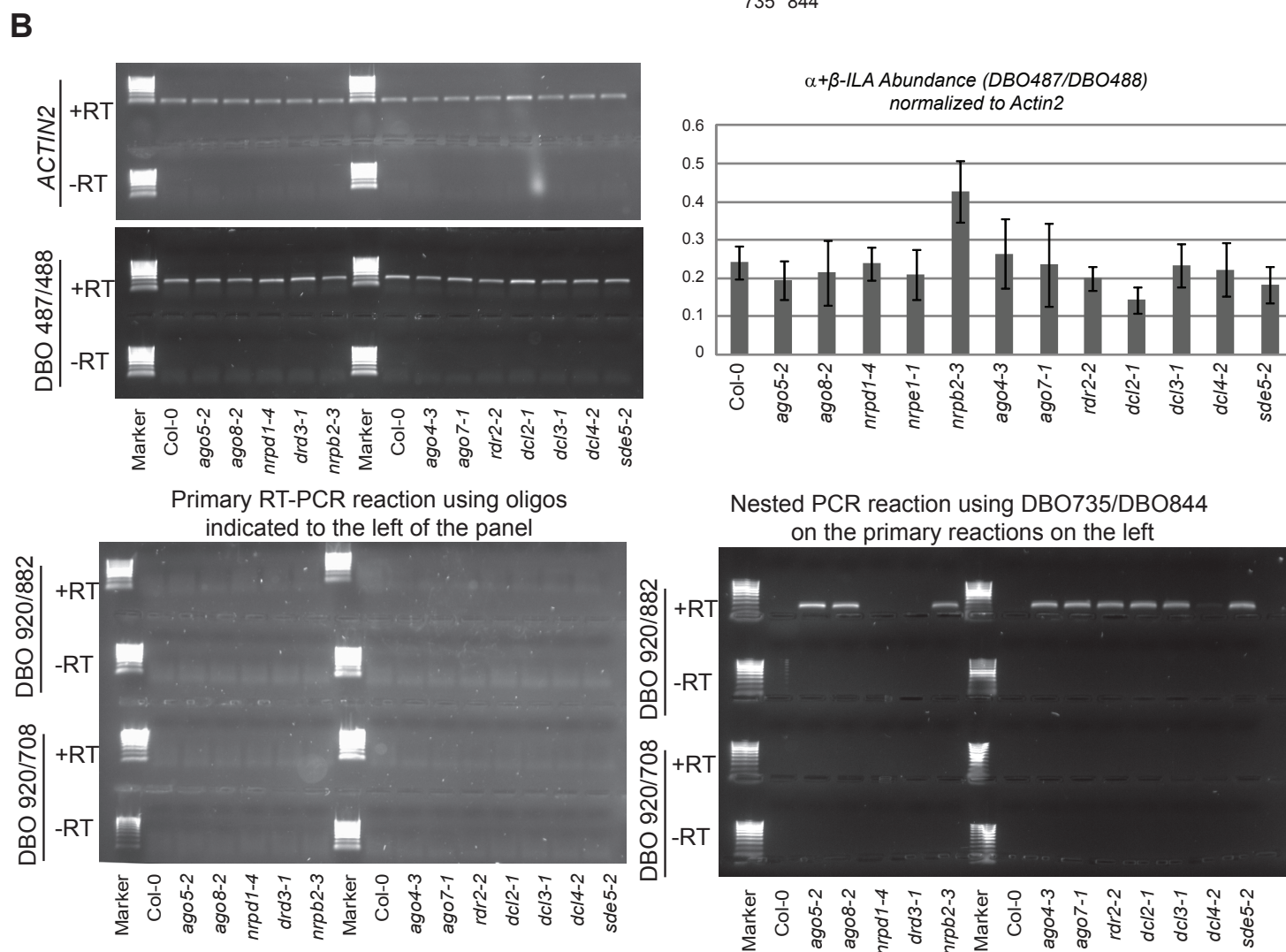

Supplement: Figure S7 — Transcript abundance changes of ILA in mutants with various RITA epigenetic states. (a) Locus structure of ILA and RITA. Upper panel depicts the orientation of α - and β-ILA relative to RITA. Lower panel is a schematic of the RNA transcripts present at the locus. Open boxes represent coding regions; gray boxes represent UTRs; RITA is depicted in black. Labelled arrows correspond to the oligos used in RT-PCR reaction shown in (b). (b) RT-PCR of ILA transcripts. ACT2 and α+β-ILA (most likely to be composed of mainly α -ILA) as measured by DBO487/DBO488do not significantly differ amongst the RNA silencing mutants tested. A nested PCR reaction using DBO735/DBO844on a primary PCR reaction designed only to amplify β-ILA transcripts demonstrated an increase in β-ILA in the mutants where methylation is lost at RITA. No amplification was observed in a primary reaction (DBO920/DBO882) or when the oligos were present outside of the mapped β-ILA transcript region (DBO920/DBO708). (PDF) [file pone.0045242.s007.pdf]
