# Supplementary material for: Metastable Differentially Methylated Regions within Arabidopsis Inbred Populations Are Associated with Modified Expression of Non-Coding Transcripts
Source: PLoS One. 2012 Sep 20;7(9):e45242. doi: 10.1371/journal.pone.0045242 (PMC3447930; doi:10.1371/journal.pone.0045242)
Supplement: Figure S6 — Coding predictions for RITA and MRD1 . (a) The RITA RNA transcript has two Open Reading Frames (ORFs). The predicted number of amino acids appears below the ORF. Predicted homologous proteins are listed for ORF2 with accession, description and E value. (b) The MRD1 transcript contains 3 ORFs. The number of amino acids predicted for each ORF is listed below the schematic. Blast P results including accession, description, and E value are listed for each of the hits. (PDF) [file pone.0045242.s006.pdf]

Figure S6

A

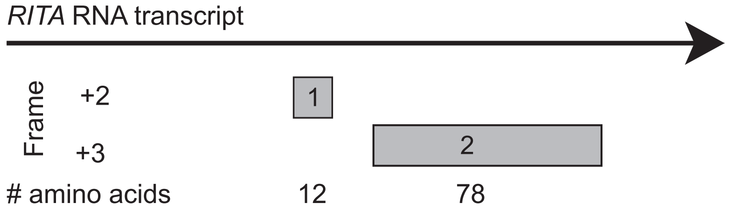

BLAST P results for ORF2

Accession	Description	Total Score	Query Coverage	E value
XP_846961.1	hypothetical protein [*Trypanosoma brucei* TREU927]>gb|AAX70328.1	33.5	63%	8.2
ZP_01463987.1	phosphoribosylformylglycinamidine synthase [*Stigmatella aurantiaca* DW4/3-1] >gb|EAU65257.1T	33.1	70%	10.0

B

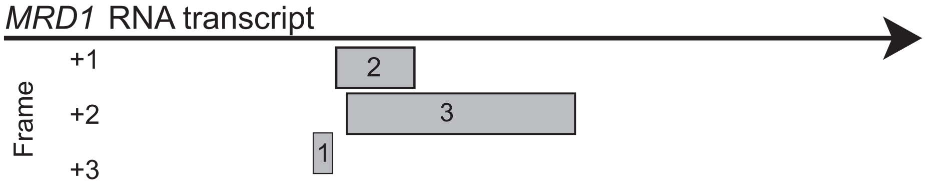

ORF1 - 5 amino acids - MERVW
ORF2- 32 amino acids - MGNYDSRRRQQIWWISSSVSSRRYLSSDEPPW
ORF3 - 120 amino acids

BLAST P results for ORF3

Accession	Description	Total Score	Query Coverage	E value
XP_002887100.1	hypothetical protein ARAYLDRAFT_475803 [*Arabidopsis lyrata* subsp. lyrata] >gb|EFH63359.1	130	100%	7e-29
NP_195929.1	hypothetical protein [*Arabdiopsis thaliana*]>emb|CAB86076.1|	122	65%	2e-26
ZP_08108626.1	hypothetical protein HMPREF9475_03490 [*Clostridium symbiosum* WAL-14673] >gb|EGB17427.1|	37	68%	0.88
ZP_08092817.1	hypothetical protein HMPREF9474_04568 [*Clostridium symbiosum* WAL-14163] >gb|EGA91609.1|	37	68%	0.89
ZP_08176587.1	adenine specific DNA methylase Mod [*Xanthomonas vesicatoria* ATCC 35937] >gb|EEGD11138.1|	36.2	60%	1.3
NP_636443.1	DNA methylase [*Xanthomonas campestris* pv. campestris str ATCC 33913] >gb|AAM40367.1|	35.8	60%	1.8
ZP_02389774.1	putative modification methylase [*Burkolderia thailandensis* Bt4]	35.4	60%	2.5
ZP_02353897.1	putative modification methylase [*Burkolderia oklahomenensis* EO147]	35.4	60%	2.5
YP_703251.1	aspartate aminotransferase [*Rhodococo justii* RHA1] >gb|ABG95093.1|	35	36%	3.0
NP_881682.1	putative modification methylase [*Bordetella pertussis* Tohama I] >emb|CAE43384.1|	34.3	60%	6.1
YP_002780024.1	aspartate aminotransferase [*Rhodococo opacus* B4] >dbj|BAH51079.1|	33.9	36%	7.9
